# Meta-analysis of associations between neutrophil-to-lymphocyte ratio and prognosis of gastric cancer

**DOI:** 10.1186/s12957-015-0530-9

**Published:** 2015-03-26

**Authors:** Jing Chen, Dongsheng Hong, You Zhai, Peng Shen

**Affiliations:** Department of Medical Oncology, The First Affiliated Hospital of College of Medicine, Zhejiang University, 79 Qingchun Road, Hangzhou, 310003 People’s Republic of China; Department of Pharmacy, The First Affiliated Hospital of College of Medicine, Zhejiang University, 79 Qingchun Road, Hangzhou, 310003 People’s Republic of China

**Keywords:** Gastric cancer, Neutrophil-to-lymphocyte ratio, Meta-analysis, Prognostic

## Abstract

**Background:**

The prognostic role of inflammation indices, such as the neutrophil-to-lymphocyte ratio (NLR) in gastric cancer (GC) remains controversial. We conducted a meta-analysis to determine the predictable value of NLR in the clinical outcome of GC patients.

**Methods:**

We searched Embase, PubMed and the Cochrane Library database for relevant randomised controlled trials. Statistical analyses were conducted to calculate the hazard ratio (HR) and 95% confidence intervals of overall survival (OS) and progression-free survival (PFS) using either random-effect or fixed-effect models according to the heterogeneity of the included studies. An analysis was carried out based on data from nine studies to evaluate the association between NLR and OS in patients with GC.

**Results:**

Our analysis indicated that elevated pre-treatment NLR predicted poorer OS (HR: 2.16, 95% CI: 1.86 to 2.51, *P* < 0.001) and PFS (HR 2.78, 95% CI: 1.95 to 3.96; *P* < 0.00001) in patients with GC. Over a 3-year follow-up period, high NLR was a significant predictor of poor outcomes at year 1 (HR 1.99; 95% CI: 1.39 to 2.85; *P* = 0.0002), year 2 (HR 2.24; 95% CI: 1.69 to 2.97; *P* < 0.00001) and year 3 (HR 2.80; 95% CI: 1.98 to 3.96; *P* < 0.00001).

**Conclusions:**

Elevated preoperative NLR is associated with poorer rates of survival in GC patients and may play a role in GC surveillance programmes as a means of delivering more personalised cancer care.

**Electronic supplementary material:**

The online version of this article (doi:10.1186/s12957-015-0530-9) contains supplementary material, which is available to authorized users.

## Background

Despite its declined incidence in recent decades, gastric cancer (GC) remains a major health problem around the world [1]. It is the fifth most common cancer worldwide, with about one million (952,000) new cases diagnosed annually, and it was the third leading cause of cancer deaths (723,000 deaths) in 2012, according to the World Health Organization’s GLOBOCAN database [2]. Despite rapid developments in surgery, chemotherapy and molecular therapy in the recent years, the clinical outcome of GC is still not promising. This is mainly due to local tumour recurrence or distal metastasis. The progression of tumour staging systems for GC can be used to predict prognosis and guide patient therapy; however, heterogeneity of prognosis still exists among patients in the same stage [3]. It is increasingly recognised that variations within clinical outcomes in cancer patients are influenced by not only the oncological characteristics of the tumour but also the host-response factors [4]. The possibility of combining multiple, clinically available host- and tumour-related factors is of great interest, as it might serve as an excellent basis for clinical decision-making, treatment planning and establishing follow-up schedules.

Several articles have suggested that GC could induce inflammation in the host [5], revealing a close relationship between these tumours and chronic inflammation [6]. This inflammatory response reflects a non-specific response to tumour hypoxia tissue injury and necrosis [7,8]. The complex and diverse neuroendocrinological and haemopoetic changes that occur during inflammation are thought to be responsible for the diminishment of the immune response and the increase in tumour proliferation [9]. Work has been undertaken to identify components of this inflammatory response that might identify patients at risk of poorer outcomes. Generally speaking, lymphopenia is the surrogate of an impaired cell-mediated immunity, whereas neutrophilia is a response to systematic inflammation [10]. NLR calculated as neutrophil counts divided by lymphocyte counts, is suggested as a marker for general immune responses to various stress stimuli [11]. It is thought that a high NLR increased systemic inflammatory in the host and associated with a poorer prognostic outcome. Emerging evidence shows that NLR is a prognostic and predictive biomarker in patients with some cancers, including breast cancer, hepatocellular carcinoma and colon cancer [12-14]. Elevated NLR levels in GC patients may be independent predictors of poor OS [15]. Due to variance in study design and sample size, some authors did not agree with the prognostic value of NLR in gastric cancer [16]. The direct impact of the NLR level on patient survival and tumour clinicopathological variables remains inconclusive.

This study aimed to systematically review the literature and use meta-analysis to evaluate the prognostic utility of NLR in these patient groups. Additionally, the relationship between NLR and clinicopathological factors was investigated.

## Methods

### Search strategy

The study was executed and reported in accordance with the Preferred Reporting Items for Systematic Reviews and Meta-Analyses (PRISMA) statement and was registered with the International Prospective Register of Systematic Reviews (PROSPERO). All studies published between 2004 and 2013 (without language restriction) reporting on NLR and prognosis in patients undergoing GC treatment were identified. The MEDLINE, EMBASE and Cochrane databases were searched using the following medical subject heading (MeSH) terms or free text: (‘neutrophil’ or ‘neutrophils’) and (‘lymphocyte’ or ‘lymphocytes’) and (‘clinical trial’ or ‘randomised controlled trials’ or ‘study’ or ‘prospective study’ or ‘randomised controlled trials as topic’). The ‘related articles’ function and the reference lists of each of the identified publications were used to widen the literature search. All relevant review articles were also screened.

#### Data extraction

Two reviewers (CJ and HD) independently assessed the articles. Relevant data were extracted without cross-referencing, and any conflicts in data extraction or quality assessment were resolved by a third reviewer (ZY) before analysis. The inclusion/exclusion criteria and outcome measures are described below (Additional file 1: Table S1). The quality of the included studies was assessed according to the Scottish Intercollegiate Guidelines Network (SIGN) [17].

### Inclusion and exclusion criteria

For inclusion in this analysis, studies had to compare survival in GC patients with ‘high’ pre-treatment NLR (HNLR) *versus* ‘low’ pre-treatment NLR (LNLR). Studies had to report the outcomes of interest mentioned below and comprise an adult patient group (aged ≥18 years). Studies were included if NLR was calculated using routine full blood count analysis performed preoperatively for primary GC before the initiation of chemotherapy for advanced GC or before pre-treatment for GC. Studies were excluded if they included patients with other cancers from which the GC group could not be separated or if they reported a previously published data set.

### Outcome measures

OS was the main outcome of interest for studies with patients undergoing curative resection of primary GC. PFS was the main outcome of interest for studies with patients undergoing palliative chemotherapy for advanced GC. PFS was defined as an absence of progression and increase in volume of the primary GC or metastatic disease over the follow-up period. The following data elements were extracted: study type number of patients, stage of GC, treatment type, follow-up (months), timing of the NLR recorded, cut-off value used to determine ‘high’ *versus* ‘low’ NLR and number of patients in each group.

### Statistical analysis

The logarithm of the hazard ratio (HR) with 95% confidence interval (CI) was used as the primary summary statistic [18]. To estimate HR and its variance, annual mortality rates, survival curves, number of deaths or percentage of freedom from death were extracted from the study directly or required additional calculation depending on the method of data being presented. Calculation of the logarithm of the HRs and their 95% CI was also performed yearly for the first 3 years after treatment [19]. Meta-analysis of the data was conducted using a random-effects model. Publication bias was explored graphically with funnel plots to detect asymmetry and any outliers. Inter-study heterogeneity was assessed using the square statistic and the *I*^2^ value to measure the degree of variation not attributable to chance alone. This was graded as low (*I*^2^ < 25%), moderate (*I*^2^ = 25 to 75%) or high (*I*^2^ > 75%). Calculations were performed by CJ and verified by HD. This study was performed in line with Cochrane recommendations, following PRISMA guidelines and using the statistical software Review Manager Version 5.1 (The Cochrane Collaboration, Oxford, UK).

## Results

### Search outcomes

**Figure 1:**
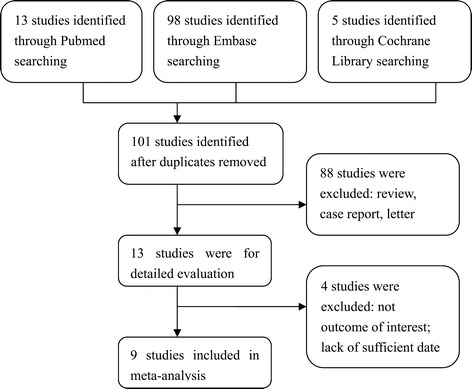
**Flow chart demonstrating process of study selection.**

After removal of the duplicates, 116 citations were identified during the reported systematic literature search. Of these, 103 were excluded through abstract reviews, leaving 13 articles. Of these 13, 3 were excluded because they did not provide enough data for estimating the HR and 95% CI. One other study was excluded because it reported the prognostic value of the inflammation index constructed by NLR and another index (c-reactive protein) but failed to present NLR-specific data. Therefore, nine studies published between 2007 and 2013 were included in our meta-analysis. A flowchart of the literature search is shown in Figure 1.

The nine selected studies included 3,709 patients with 36.5% classified as HNLR. The cut-off value for HNLR was <3 in four studies [20-23], 3 ≤ to ≤4 in four studies [15,24-26] and ≥5 in one study [27]. Six studies assessed patients undergoing resection of the primary GC, and three studies reported patients undergoing palliative chemotherapy for GC. NLR was calculated based on pre-treatment laboratory data using white blood cell (WBC) differentiated counts in all of the studies. Three of these cohorts enrolled <200 patients and six cohorts enrolled >200 patients. HR and 95% CI were reported directly in the original literature in six of the enrolled cohorts. In three of the nine cohorts, HR was produced by multivariate analyses. A summary of the study characteristics is presented in Table 1

**Table 1:** **Main characteristics of all the studies included in the meta-analysis**

**First author**	**Publish time**	**Age (years)**	**Number of patients; stage of TNM**	**Treatment arm**	**NLR cutoff (HNLR/LNLR)**	**Follow-up (months) (median and range)**	**Study quality**
Lee S	2013	55 ± 12.4^a^	7/22/41/104; I to IV	Palliative/adjuvant chemotherapy	3 (62/112)	14.9 (1.0 to 47.9)^b^	6
Lee DY	2013	57 (23 to 89)^b^	110/35/62/3; I to IV	Adjuvant chemotherapy	2.15 (50/164)	NR	5
Jin H	2013	60 (37 to 77)^b^	36/8; III to IV	Neoadjuvant chemotherapy	2.5 (24/22)	NR	5
Dirican A	2013	58 (30 to 86)^b^	6/20/105/105; I to IV	Palliative/adjuvant chemotherapy	3.8 (89/147)	60	5
Wang DS	2012	<65/230 ≥ 65/94	324; III	Adjuvant chemotherapy	5 (156/168)	39.9 (23.77 to 57.43)^b^	6
Jeong JH	2012	52.5 (28 to 82)^b^	104; IV	Palliative chemotherapy	3 (55/49)	11.9 (10.2 to 13.5)^b^	6
Mohri Y	2010	63.4 (32 to 87)^b^	232/57/68; I to III	Adjuvant chemotherapy/ neoadjuvant chemotherapy	2.2 (130/227)	68 (1 to 70)^b^	5
Shimada H	2010	65 (26 to 89)^b^	584/132/153/159; I to IV	Adjuvant chemotherapy	4 (127/901)	23 (12 to 84)^b^	5
Yamanaka T	2007	<60/493 ≥ 60/727	1220; IV	Palliative chemotherapy	2.5 (664/576)	15.6	6

NLR, neutrophil-to-lymphocyte ratio; HNLR, high pretreatment NLR; LNLR, low pretreatment NLR; NA, not applicable. ^a^Data as mean ± SD. ^b^Data as mean (range).

### NLR and OS in GC

**Figure 2:**
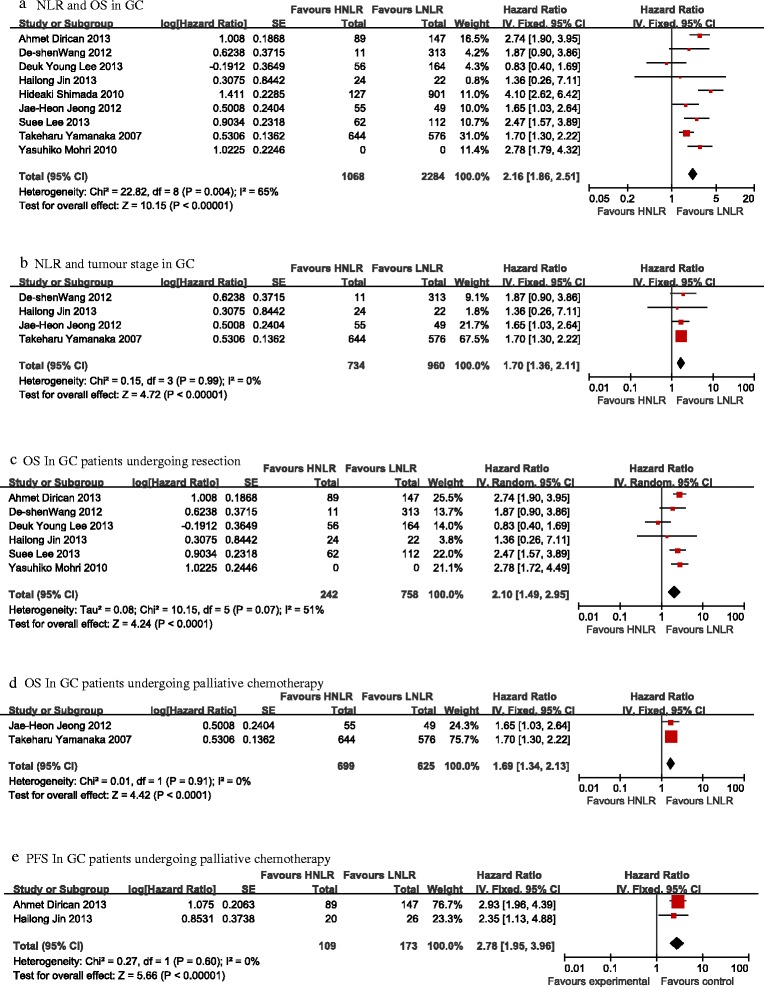
**Forest plots of survival in pretreatment HNLR** ***versus*** **LNLR patients for studies. (a)** NLR and OS in GC. **(b)** NLR and tumour stage in GC. **(c)** OS In GC patients undergoing resection. **(d)** OS In GC patients undergoing palliative chemotherapy. **(e)** PFS In GC patients undergoing palliative chemotherapy.

There were nine cohorts presenting data for pre-treatment NLR and OS in GC patients. However, with heterogeneity (*I*^2^ 65%, *P* = 0.004), the pooled HR of 2.16 (95% CI: 1.86 to 2.51, *P* < 0.001) showed that patients with elevated NLR were expected to have shorter OS after treatment. The forest plot for this is shown in Figure 2a.

### NLR and tumour stage in GC

Four studies presented OS data on NLR and stages III to IV in GC. The combined HR of 1.70 (95% CI: 1.36 to 2.11 *P* < 0.00001 Figure 2b) with no heterogeneity (*I*^2^ = 0%, *P* = 0.99) suggested that patients with elevated NLR showed a propensity towards an advanced TNM stage. Stage III to IV GC patients were significantly worse in the HNLR group.

### GC patients undergoing resection of the primary lesion

OS among the six studies included in this subgroup analysis showed a significant survival disadvantage in the HNLR group (HR 2.10, 95% CI: 1.49 to 2.95, *P* < 0.0001). There was an overall moderate but not statistically significant level of heterogeneity between the individual studies (*I*^2^ = 51%, *P* = 0.07). Figure 2c represents the forest plot for this analysis.

### Stage IV GC patients undergoing palliative chemotherapy

GC patients undergoing palliative chemotherapy showed significantly worse OS (HR 1.69, 95% CI: 1.34 to 2.13, *P* < 0.0001) and PFS (HR 2.78, 95% CI: 1.95 to 3.96, *P* < 0.00001) in the HNLR group when considering the two studies included in this subgroup. Figure 2d,e represents the forest plot for this analysis. The heterogeneity level between these two studies was low and insignificant: *I*^2^ = 0%, *P* = 0.91 and *I*^2^ = 0%, *P* = 0.60, respectively.

### Impact of follow-up duration on HR for survival in GC patients

**Figure 3:**
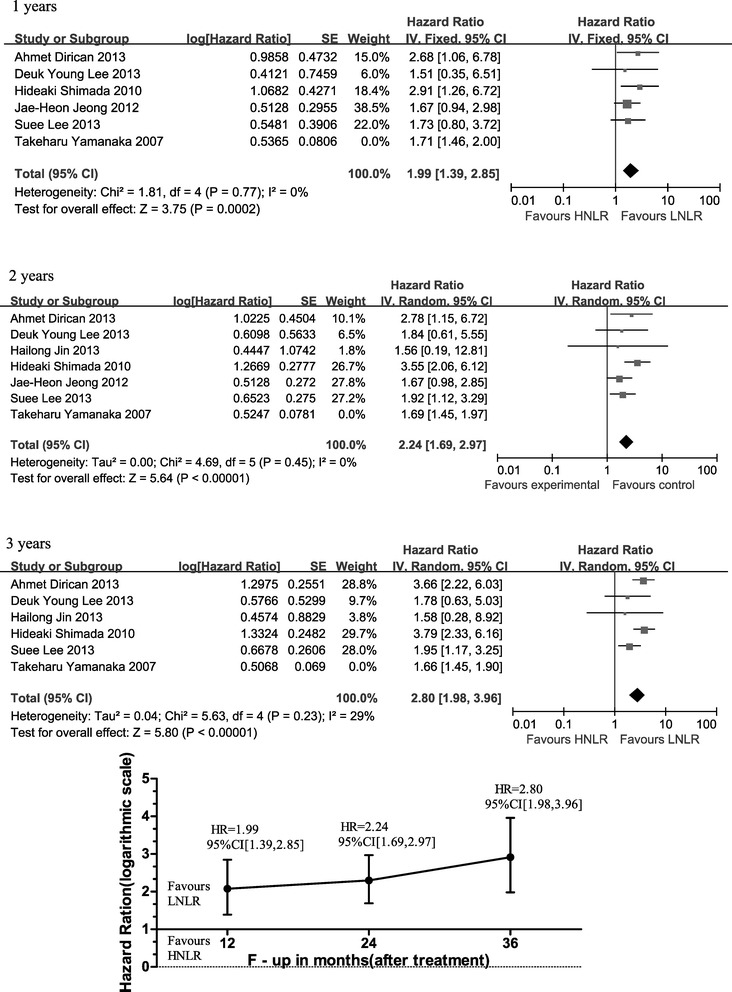
**Forest plots demonstrating the 1-, 2- and 3-year OS HR for all studies included, and linear representation of follow-up trends on a yearly basis.**

HR for survival of HNLR *versus* LNLR after the first year of follow-up could be calculated for six studies [15,20,23-26], for seven studies after the second year of follow-up [15,20,22-26] and for six studies after the third year of follow-up [15,20,22-25]. Figure 3 represents the forest plot for the three time points, as well as a cumulative graph of the HRs. Survival for the first year and years 2 or 3 of follow-up were significantly different between groups (HR 1.99, 95% CI: 1.39 to 2.85, *P* = 0.0002): at years 2 (HR 2.24, 95% CI: 1.69 to 2.97, *P* < 0.00001) and 3 (HR 2.80, 95% CI: 1.98 to 3.96, *P* < 0.00001). HR for survival was significantly worse for the HNLR *versus* LNLR groups.

### Sensitivity analyses

A single study involved in the meta-analysis was deleted each time to unveil the influence of the individual data set on the pooled HRs (or ORs); the corresponding pooled HRs (or ORs) were not materially changed (data not shown).

### Heterogeneity assessment

Bias exploration funnel plots were created for combined and subgroup analysis for OS to visually assess the publication bias (Additional file 2: Figure S1). They demonstrated symmetry. Exclusion of the outliers did not significantly alter the results.

## Discussion

The TNM staging system which mainly focuses on the biological behaviour and presentation of the tumour itself acts as the foundation for subdividing GC patients and determining suitable treatments. However, staging systems are inadequate methods to precisely predict prognosis and appropriately guide clinical practice because patients at the same stage may have various clinical outcomes. The introduction of the laboratory index as a supplementary item to the current tumour staging system has significant potential to help practitioners create personalised treatment strategies. So far, the prognostic significance of the marker of systematic inflammatory reaction to solid tumours has received relatively little attention in the pursuit of tumour-based molecular evaluations of outcome.

A simple marker of systemic inflammation is NLR. Elevated NLR has recently been shown associated with poorer prognosis in patients with various types of malignant tumours [12-14]. The cut-off value for defining high NLR has not been unified in our meta-analysis. Meanwhile, some authors defined cut-off value as 2, 3, 4 or 5 by analysing the ROC curve or just arbitrarily, which led to between-study heterogeneity [28,29]. However, the NLR’s prognostic value was not affected, as the majority of the subgroup analysis did not change the results substantially. In addition, sensitivity analysis did not draw different conclusions from the pooled estimate. A future large sample study is needed to give a definitive cut-off value of NLR with good sensitivity and specificity.

The present study analysed the association between NLR and prognosis as well as the clinicopathological parameters in GC. We showed that increased pre-treatment NLR, a systemic inflammation-based prognostic score, could predict OS in patients undergoing primary resections for GC and in patients undergoing palliative chemotherapy. We also found that NLR has a role in predicting PFS in patients undergoing palliative treatments for GC. HNLR was not only associated with a poorer prognosis when all patient groups were combined but also during subgroup analysis. Furthermore, subgroup analysis confirmed these findings in each of the groups and produced a significantly lower level of heterogeneity, as was expected. There was also a significant association between NLR and grade of tumour stage. Taking all these into consideration, NLR is a promising prognostic marker to assist in the clinical decision-making process regarding GC treatment and outcomes. Cancer-related inflammation has been shown to have adverse effects on cancer prognosis.

Our results have also identified a potential role for NLR as a predictor of survival during post-therapy follow-up particularly from between 1 and 3 years post-treatment. Paramanathan [30] found that high NLR correlated with worse long-term outcomes following curative intent surgery on solid tumours. Median 5-year OS for higher NLR compared to lower NLR was 35.8% *versus* 70.1%. These results provide evidence to support the hypothesis that the NLR potentially represents a simple and robust measurement of prognosis. However, this preliminary finding requires further investigation before NLR can be recommended for inclusion in GC surveillance programmes. It needs to be validated in larger prospective studies for it to be useful in risk stratification.

To date, there has been one previous meta-analysis examining the role of NLR in predicting overall survival and PFS [31]. This study had similar aims to our own study and produced similar results, but differed in several key regards. First, it selected more patients for the meta-analysis (3,709 *versus* 2,952), making it significantly more powerful. Second, the subgroups of patient treatment type used in the previous study were not as well defined. Third, the subgroups of patient tumour stage used by the previous study were also not as well defined. Finally, our study also investigated the role of NLR in predicting survival as part of a GC surveillance programme. Based on our results, the significant value of NLR is that it can identify patients at high risk of disease progression and death as a clinically convenient and useful biomarker. Thus, it not only provides guidance for clinical follow-up care but also has the potential to be a stratification factor or a selection criterion in randomised clinical trials for metastatic GC.

The reason for the association between elevated NLR and progression of tumour growth is not fully understood. One possible mechanism for this association is that tumour-associated neutrophils remodel the tumour microenvironment resulting in the release of MMP family members, which act on pro-inflammatory cytokines, chemokines and other proteins to regulate diverse aspects of inflammation. This plays an active role in maintaining tumour-promoting inflammation [32]. In addition, neutrophil-derived reactive oxygen species further decrease the adhesion-promoting properties of the extracellular matrix and, via activation of nuclear factor (NF)-kB and STAT3, inhibit apoptosis of the tumour cells. These events result in accelerated tumour progression, invasion of the surrounding tissues, angiogenesis and often metastasis [33-35]. Finally, T lymphocyte cells are the primary cells responsible for direct recognition and killing of tumour cells. The long life of memory T cells (Tm) determines their crucial role in carcinogenesis and carcinogenic progression. Tm in peripheral blood from GC patients was statistically lower than those of healthy donors. The gastric cancer patients in stages III to IV had significantly lower levels of Tm compared to patients in stages I to II. Therefore, reduction of Tm may be related to immunodeficiency of gastric cancer [36].

There are a number of limitations of our study, many of which also apply to meta-analysis research in general. This study was limited to analysing studies published in English, so publication bias cannot be excluded. Heterogeneity among these studies was also relatively large; this might be caused by the fact that they were conducted in different countries or used patients with different histological types of cancer among other factors. Randomised controlled trial research is not appropriate in this setting, but research with larger patient groups is required so that a more robust subgroup analysis can be performed.

Body composition changes especially muscle-mass depletion have been associated with the systemic inflammatory response (SIR) in GC patients, and this relationship might indicate the mechanism by which reduced muscle mass is associated with worse outcomes. Inflammation generates not only a cancer-promoting microenvironment but also systemic changes in the host that favour cancer progression. We believe that this meta-analysis provides good evidence for an altered SIR, expressed as NLR, acting as a promoter in the fatal progression of GC. Modifying a patient’s SIR may become as important a therapeutic target as the tumour itself. Whether preoperative NLR can be altered before intervention and thereby influence long-term outcomes remains to be established. Preoperative administration of corticosteroids in patients undergoing surgery for cancer is associated with a reduction in post-operative morbidity [37]. This observation may be due to the alteration of the inflammatory response to surgery [37-39]. Also, studies suggest that non-steroidal anti-inflammatory drugs (NSAIDs) have a preventative effect against the development and progression of GC [40].

NLR is an easily measurable inflammatory biomarker. Our results demonstrate that an elevated NLR is associated with worse OS and a lower disease-free interval in patients with GC. Our study therefore highlights the importance of NLR as a predictor of survival during post-therapy follow-up. To date, no specific therapies or interventions to modify a high NLR exist. Interventions to modify pre- and post-operative inflammatory responses and to modulate the immune response may prove beneficial in improving long-term cancer outcomes. The ability of NLR to predict transition to and toxicity from therapies is of particular interest, and future studies should aim to address these possibilities.

## Conclusions

Elevated preoperative NLR is associated with poorer rates of survival in GC patients and may play a role in GC surveillance programmes as a means of delivering more personalised cancer care.

## Additional files

Additional file 1: Table S1. Quality scores of the studies included in this systematic review. Additional file 2: Figure S1. Begg’s funnel plot for publication bias of all studies on overall survival.

## Abbreviations

GC: Gastric cancer
HNLR: High NLR
HR: Hazard ratio
LNLR: Low NLR
NLR: Neutrophil-to-lymphocyte ratio
NSAIDs: Non-steroidal anti-inflammatory drugs
OS: Overall survival
PFS: Progression-free survival
SIR: Systemic Inflammatory response
WBC: White blood cell
