# Supplementary figures and images for: Meta-analysis of associations between neutrophil-to-lymphocyte ratio and prognosis of gastric cancer
Source: World J Surg Oncol. 2015 Mar 26;13:122. doi: 10.1186/s12957-015-0530-9 (PMC4379945; doi:10.1186/s12957-015-0530-9)

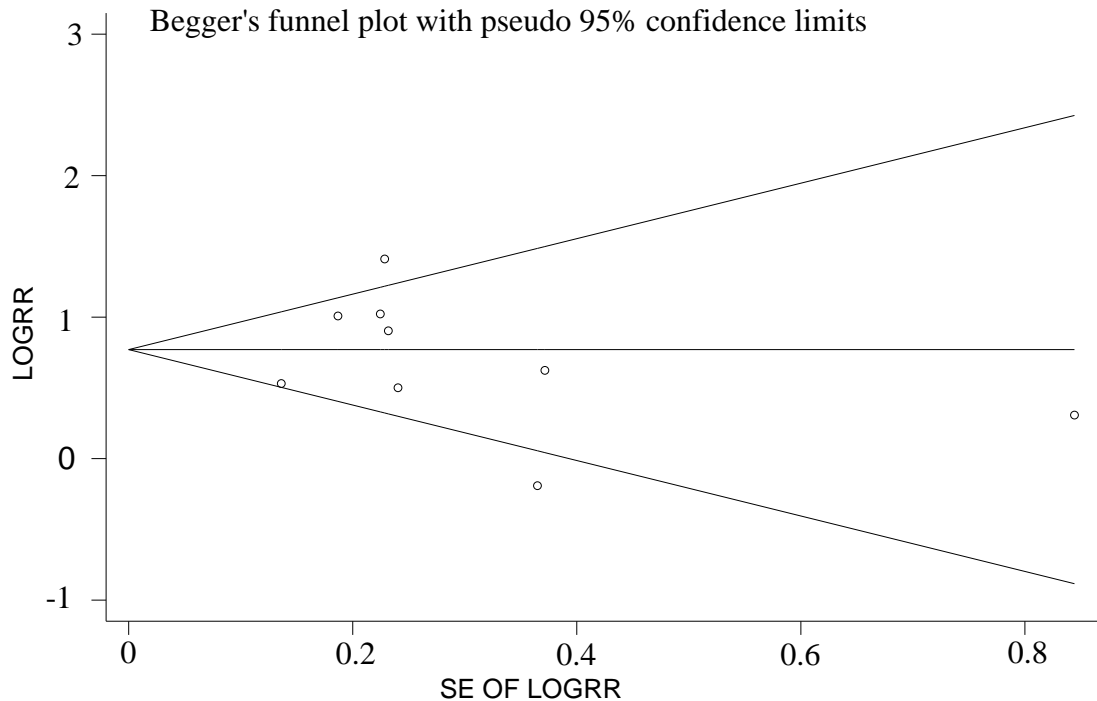

Fig. S1 Begg's funnel plot for publication bias of all studies on overall survival.

Supplement: Additional file 2: Figure S1. — Begg’s funnel plot for publication bias of all studies on overall survival. [file 12957_2015_530_MOESM2_ESM.pdf]
