# Supplementary material for: Meta-analysis of associations between neutrophil-to-lymphocyte ratio and prognosis of gastric cancer
Source: World J Surg Oncol. 2015 Mar 26;13:122. doi: 10.1186/s12957-015-0530-9 (PMC4379945; doi:10.1186/s12957-015-0530-9)
Supplement: Additional file 1: Table S1. — Quality scores of the studies included in this systematic review. [file 12957_2015_530_MOESM1_ESM.pdf]

Table S1. Quality scores of the studies included in this systematic review

Year (ref)	Randomized	Blinded	Prospective study	Control group	Inclusion Criteria defined	Intervention defined	Outcome defined	Baseline characteristics similarity	Intent to treat analysis	Details drop-outs deaths	Total score
2013(22)	N	N	N	Y	Y	Y	Y	Y	N	Y	6
2013(23)	N	N	N	Y	Y	Y	Y	N	N	Y	5
2013(24)	N	N	N	Y	Y	Y	Y	N	N	Y	5
2013(25)	N	N	N	Y	Y	Y	Y	N	N	Y	5
2012(26)	N	N	N	Y	Y	Y	Y	Y	N	Y	6
2012(27)	N	N	N	Y	Y	Y	Y	Y	N	Y	6
2010(15)	N	N	N	Y	Y	Y	Y	N	N	Y	5
2010(21)	N	N	N	Y	Y	Y	Y	N	N	N	5
2007(20)	N	N	N	Y	Y	Y	Y	Y	N	Y	6
Mean score											
